# Conference Report: INTERNATIONAL WORKSHOP ON SEMICONDUCTOR CHARACTERIZATION: PRESENT STATUS AND FUTURE NEEDS Gaithersburg, MD January 30 – February 2, 1995

**DOI:** 10.6028/jres.100.053

**Published:** 1995

**Authors:** D. G. Seiler, T. J. Shaffner

**Affiliations:** Semiconductor Electronics Division, National Institute of Standards and Technology, Gaithersburg, MD 20899-0001; Texas Instruments, Inc., Dallas, TX 75265

## 1. Introduction

The International Workshop on Semiconductor Characterization: Present Status and Future Needs was held at the National Institute of Standards and Technology from January 30 to February 2, 1995. This comprehensive, “world-class” workshop was dedicated to summarizing major issues and giving critical reviews of important semiconductor characterization techniques that are useful to the semiconductor industry. Because of the increasing importance of in-line and in-situ characterization methods, the workshop placed a strong emphasis on these methods.

Specific goals of the workshop were: (1) to provide a forum in which measurements and technical issues of current and future interest to the semiconductor industry could be reviewed, discussed, critiqued, and summarized; (2) to demonstrate and review important applications for diagnostics, manufacturing, and in-situ monitoring and control in real-time environments; (3) to provide a silicon integrated circuit process and materials-based view of requirements for off-line, in-line, and in-situ analysis and metrology; (4) to focus attention on the critical and unique requirements related to compound semiconductor materials and devices; and (5) to act as an important stimulus for new progress in the field by providing new perspectives.

The workshop provided a concise and effective portrayal of industry characterization needs and the problems that must be addressed by industry, government, and academia to continue the dramatic progress in semiconductor technology.

## 2. Semiconductors—The Backbone of All Modern Microelectronics and Optoelectronic Devices

Semiconductors and their applications are one of the greatest scientific and technological breakthroughs of this century. Consider the impact of microelectronic components used in computers, entertainment equipment, automotive electronics, medical instrumentation, telecommunications, space technology, television, radio, and manufacturing technologies. Almost every factory, hospital, office, bank, school, or household contains transistors, microprocessors, and other semiconductor devices. Semiconductor characterization is an indispensable enabler of all modern microelectronics and optoelectronic circuits, and is in the critical path for maintaining the steady decline in cost-per-function of silicon integrated circuit technology. It is also helping to drive new developments in compound semiconductor materials and devices (III–V and II–VI).

Semiconductor materials and devices must continually meet more stringent requirements as the density and performance of semiconductor devices increase. The purity, perfection, and cleanliness required of the materials; the ultra-small dimensions of the devices; and the device properties themselves require measurements to a higher precision and with a resolution and sensitivity that pushes these techniques to their very limit. In addition, new techniques associated with analysis of process chemicals, control of process steps, and characterization of packages are critically needed. Because the materials and devices themselves exhibit a rich variety of properties, an increasingly wide range of measurement techniques has evolved to meet industry’s needs. Meeting the demands for large-scale, complex, integrated circuits will continue to require technological advances in materials, processing, circuit design, characterization, testing, and standards.

Compound semiconductors are also being used in a variety of structures for light-emitting diodes, laser diodes, far-infrared detectors, and microwave devices. In addition to being used in their bulk and natural forms, these semiconductor materials are used in artificially created structures such as superlattices and heterostructures. Various compounds are mixed to produce structures in which properties such as the bandgap have been engineered to have specific values. Such structures and devices have unique applications in wireless communications, optical communications, visible light sources, and imaging, all of which are critical for information technologies. They also enable functions and enhanced performance that cannot be equaled by silicon-based technologies.

International economic competition is pressing the U.S. semiconductor industry to promote precompetitive cooperation among government, industry, and universities. A unified viewpoint fosters efficient use of government and university resources and effective planning for future activities. In 1992, the Semiconductor Industry Association (SIA) published a consensus view of the requirements for the manufacture of future silicon-based integrated circuits in a document known as the *1992 SIA Roadmap*. Here, key industry, government, and university technologists described a strategy for meeting materials, process, tool, and factory requirements for future IC manufacturing. The document was updated 2 years later as the *National Technology Roadmap for Semiconductors (NTRS)* [1]. In each, the importance of metrology was emphasized.

## 3. Role of Semiconductor Characterization and Metrology

A good description of materials characterization considers it as an integral part of process development and manufacturing. The Office of Science and Technology Policy 1993 fiscal year program booklet describes materials characterization as a wide range of interdisciplinary activities that determine the structure, composition, properties, and performance of materials, and the relationships among these [2]. Characterization measurements address quality assurance of incoming materials, wafer screening methods, control and monitoring of equipment and manufacturing processes, diagnostic and failure analysis, and end device performance in light of intended design and function.

The semiconductor industry refers to measurements used in process control as *metrology*. Engineers often use the word to describe procedures, such as critical-dimension (CD) measurements, which routinely monitor lithography processes inside the clean room. Others generalize it to all in-line measurements. According to the dictionary, *metrology is the science of measurement* [3]. It should also be noted that metrology includes all aspects, both theoretical and practical, with reference to measurements, whatever their uncertainty, and in whatever fields of science or technology they occur [4]. The term *characterization* is also sometimes used interchangeably with *metrology*.

Metrology must break the status quo image of being a *non-value-added* activity. For in-situ real-time control, it can improve equipment effectiveness by reducing process time, down time, and use of test wafers, while maintaining product quality. In some cases, it is advantageous to replace metrology tools requiring test wafers with others that measure product wafers nonintrusively. In many wafer fabrication facilities (fabs), defect metrology is a key element in maintaining high yield. This includes particle and defect detection and characterization on patterned wafers using particle sensors and electrical yield testing.

In high-yield manufacturing, metrology requirements switch from fundamental measurement to verification of repetitive execution. In-situ and in-line sensors are evolving for process control, and today reach the manufacturing floor through vendors who integrate them into new equipment. Sensors add value by detecting excursions early on that might persist through the wafer fab with cumulative line yield loss.

Analytical laboratory researchers sensitive to this perspective focus their efforts on technologies that are less mature. In contrast, process development engineers sometimes stretch existing metrology too thin by pushing sophisticated instruments into a regime where they are not very effective or technically applicable. Metrology has the potential to establish a common denominator for transitions between all of the process maturity phases, and thereby reduce operational barriers.

Metrology tools applied in-line to manufacturing, and in-situ inside commercial instruments are initially developed as scientific prototypes in the R&D environment. These then evolve into stable user-friendly modules, suitable for introduction into the off-line wafer fab analytical laboratory. Further maturity brings durability and reproducibility through robust design adaptable to the wafer fab environment. Specifications and standard test methods developed by organizations such as SEMI and ASTM, and the availability of applicable certified reference materials such as NIST Standard Reference Materials (SRMs) facilitate successful transfer through these three stages. Training in relevant materials science as well as procedures of calibration are also important.

In-situ measurements are not common in volume manufacturing in the semiconductor industry today. The NTRS predicts that required factory equipment efficiency improvements will drive future incorporation of in-situ metrology. As fab managers realize the impact of a measurement tool on throughput, they will drive equipment vendors to incorporate such in-situ sensors. Ellipsometry is an example of a metrology that has progressed from R&D laboratory to an in-situ process control tool. One quarter of a century elapsed between the first R&D application of ellipsometry to semiconductor dielectric films and the demonstration of an in-situ sensor.

The progressive shrinking of ultra-large-scale integration (ULSI) circuits into the submicrometer regime and the emergence of quantum structures on the nanometer scale increase the challenge for characterization specialists. When the probe radius, depth of analysis, or contaminant level is reduced, the volume of analysis ultimately includes only several atoms of interest. In the extreme, the volume analyzed contains no atoms at all. The analyst is now confronted with a sampling problem in selecting which atoms are appropriate for the analysis. Single atom detection cannot be considered independently of the sampling problem. This issue is becoming more visible, as we realize a related problem is looming, not just in characterization, but also in the fabrication of lightly doped 3-D shallow junctions of nanometer dimensions, where only a few, or even single, dopant atoms may someday be required.

## 4. Compound Semiconductor Technology Needs Consensus-Based Planning

The NTRS established the groundwork for the creation of a metrology roadmap for silicon, which was developed through SEMATECH [1]. A comparable top-level roadmap for compound semiconductors does not yet exist, mostly because the materials systems are diverse and highly complex relative to silicon. Applications in the United States have been mostly defense related, being coordinated and executed through government funding. Also, profit margins in this country are not expected to be adequate to support a roadmap effort comparable to the NTRS. Nevertheless, segments of the industry do have defined strategies, and it is proving desirable to achieve some level of consensus in planning for future compound semiconductor technology [5].

Materials parameters generic to III–V compound semiconductors include bandgap energy, band offsets, interface diffusion and roughness, index of refraction and index dispersion, optical absorption, minority and majority carrier lifetime and mobility, and defects that influence lifetime or trapping of charge. Contactless tools include ellipsometry, light scattering, optical reflectometry, and photoreflectance, which are applied to heterostructures grown by molecular beam epitaxy (MBE) and other advanced thin-film techniques, e.g., metalorganic chemical vapor deposition (MOCVD) and metalorganic-MBE (MOMBE). These optical measurements are by nature noninvasive, and are quickly finding niches as in-situ process sensors that permit optimization of film thickness, composition, interface quality, and uniformity during growth.

Infrared detectors and imagers based on II–VI compound semiconductors are key enablers in a variety of military and space applications. HgCdTe is the material of choice for both scanning and staring focal-plane arrays. The complexity of ternary alloy systems and the stringent demands of large element detector arrays combine to increase the difficulty in working with II-VI compounds by at least an order of magnitude relative to GaAs. Material quality has reached the level of maturity required for two-dimensional focal-plane arrays based on epitaxially grown HgCdTe wafers as large as 30 cm^2^. Recent advances in surface passivation, liquid phase epitaxial (LPE) growth, and device processing have resolved many of the major materials problems encountered in the past. The principal limitation is now control of crystal point defects and impurities, especially for devices operating at 78 K or lower temperatures. There is critical demand for smaller analytical probes with higher sensitivity to resolve such problems. Incomplete understanding of physical and chemical defects continues to inhibit progress with basic II–VI materials issues.

Compound semiconductors are also candidates for high-temperature electronics applications. Although diamond offers high-thermal stability and mechanical hardness for power transistors, difficulty in growing thin films with suitable lattice-matched substrates is making it less attractive to low-cost commercial markets. Active research with compounds such as SiC and GaN is in progress. GaN has added advantages for light-emitting display applications. The characterization tools outlined above are also applicable to these developments.

Nanoelectronics is a revolutionary digital IC technology directed at continued downsizing of minimum feature size below 0.1 μm. Priority is given to eliminating long interconnects, because these are the most difficult to scale down. The approach leads to an architecture in which resonant electron quantum tunneling effects dominate, and each active element is connected only to its nearest neighbor cells (*cellular automaton*). Material systems are typically heterostructures composed of lattice-matched and doped GaAs/AlGaAs and related compounds.

Quantum structures are patterned with nanoscale resolution, which challenges even the most sophisticated small spot diagnostic probes available today. The best solutions for imaging surface detail and measuring the electrical properties of a single cell are based on scanning probe methodology. Current-voltage characteristics from a single quantum dot have been demonstrated, but are extremely difficult to reproduce. These new probes are expected to establish high-resolution capabilities applicable to future silicon circuits.

## 5. Workshop Summary

The workshop brought together over 280 scientists and engineers concerned with research, development, manufacturing, diagnostics, and other aspects of the characterization of semiconductor materials, processes, and devices. Knowledgeable people in the semiconductor field addressed the unique characterization requirements of both silicon IC development and manufacturing and compound semiconductor materials, devices, and manufacturing. Sessions on silicon ICs were based on the technology drivers in the National Technology Roadmap for Semiconductors. Additional sessions covered technology trends and future requirements for compound semiconductor applications. Also highlighted were recent developments in characterization, including in-situ, in-fab, and off-line analysis methods.

The workshop opened with introductory remarks by Arati Prabhakar, Director of the National Institute of Standards and Technology, on “Redefining the Possibilities: New Horizons in the Semiconductor World” and a Plenary Lecture by Craig Barrett, Executive Vice President and Chief Operating Officer of Intel Corporation, on “Status and Needs of the Semiconductor Industry.” These talks provided a larger context for the detailed discussions of semiconductor characterization issues in the workshop program which consisted of formal invited presentation sessions, poster sessions for contributed papers, and panel sessions.

The invited papers provided up-to-date reviews of the major issues and characterization techniques for semiconductor device research, development, and manufacturing. Poster papers, which were presented in four groups, one on each day of the workshop, emphasized new developments and improvements in characterization technology. Three panel sessions, closing each of the first 3 days of the workshop, were organized by the Semiconductor Equipment and Materials International (SEMI) to provide for multiple inputs and interactive discussion, with emphasis on the measurement equipment supplier perspective and on important issues related to the topics of the invited paper sessions. A special evening rump session explored the potential and promise of synchrotron x-ray metrology for TXRF and other analytical techniques.

The workshop was organized by a program committee chaired by David G. Seiler, National Institute of Standards and Technology. Other members of the committee were: David E. Aspnes, North Carolina State University; Ray Balcerak, Advanced Research Projects Agency; W. Murray Bullis, Materials & Metrology; Alain Diebold, SEMATECH, Inc.; Wolfgang Jantz, Fraunhofer-Institut für Angewandte Festkörperphysik; Alan Jung, SEMI; Sanjiv Kamath, Hughes Research Laboratories; Stephen S. Laderman, Hewlett Packard; Bob McDonald, Intel Corporation; William T. Oosterhuis, U.S. Department of Energy; Abbas Ourmazd, AT&T Bell Labs; Paul S. Peercy, Sandia National Laboratories; Fred H. Pollak, Brooklyn College of SUNY; John Prater, Army Research Office; Tom Remmel, Motorola; Linton G. Salmon, National Science Foundation; Tom J. Shaffner, Texas Instruments; Richard A. Singer, Institute for Defense Analyses; and William E. Tennant, Rockwell International Science Center. A NIST Advisory Committee consisting of Paul M. Amirtharaj, Frank F. Oettinger, Robert I. Scace, and James R. Whetstone also assisted in the organization of the workshop.

Chairs of the invited paper sessions were Dirk Bartelink, Hewlett-Packard; James Freedman, SRC; Len Feldman, AT&T Bell Labs; Stephanie Butler, Texas Instruments; Anne Testoni, Digital Equipment Corporation; P. B. Ghate, Texas Instruments; Paul Ho, University of Texas; Richard Brundle, Brundle Associates; Richard S. Hockett, Charles Evans & Associates; David E. Aspnes, North Carolina State University; C. Pickering, Defence Research Agency; John Prater, Army Research Office; Fred H. Pollak, Brooklyn College of SUNY; John Parsey, Motorola, Inc.; and Jerry Woodall, Purdue University. Mike Fossey, ADE Corporation; Robert I. Scace, NIST; and John C. Bean, AT&T Bell Labs chaired the panel sessions.

Sponsors of the workshop were: The Advanced Research Projects Agency, SEMATECH, the National Institute of Standards and Technology, the Army Research Office, the U.S. Department of Energy, the National Science Foundation, SEMI, the Manufacturing Science and Technology Division of the American Vacuum Society, and the Working Group on Electronic Materials of the Committee on Civilian Industrial Technologies.

In an evaluation survey conducted following the workshop, respondents indicated that the workshop had been meaningful and relevant for them because it (1) gave them insights and priorities rather than simple listings, (2) provided useful information of the capabilities of different characterization techniques, (3) gave perspectives on industrial metrology requirements, (4) explored critical needs and issues in semiconductor metrology research, (5) was relevant to technologists, and (6) brought together the international metrology community at NIST, a logical place to hold the workshop. There was considerable interest in holding another similar workshop in 2 years to 3 years in order to cover emerging techniques in semiconductor characterization as this important field develops further.

The proceedings volume, published by the American Institute of Physics, is intended to serve as a base-line reference for the characterization of semiconductors for the next decade [6]. It begins with a specially prepared paper that describes the business and manufacturing motivations for the development of analytical technology and metrology by the semiconductor industry [7]. This paper provides a comprehensive introduction to the field of semiconductor characterization and metrology, and hence to this volume.

The remainder of the volume is organized along the lines of the workshop program. The papers are grouped in seven major sections under the topics of the invited paper sessions: Drivers for Silicon Process Development and Manufacturing; Metrology Requirements for beyond 0.35 μm Geometries; Silicon Materials, Gate Dielectrics, and Process Simulation; Interconnects and Failure Analysis; Critical Analytical Methods; In Situ, Real-Time Diagnosis, Analysis, and Control; and Frontiers in Compound Semiconductors. The section on Critical Analytical Methods is further divided into eight sub-sections according to category of method. Brief summaries of the panel and rump sessions and the after-dinner remarks on Historical Perspective on Semiconductor Characterization are included as appendices.
